# CD56 Homodimerization and Participation in Anti-Tumor Immune Effector Cell Functioning: A Role for Interleukin-15

**DOI:** 10.3390/cancers11071029

**Published:** 2019-07-22

**Authors:** Heleen H. Van Acker, Zoë P. Van Acker, Maarten Versteven, Peter Ponsaerts, Daniela Pende, Zwi N. Berneman, Sébastien Anguille, Viggo F. Van Tendeloo, Evelien L. Smits

**Affiliations:** 1 Laboratory of Experimental Hematology, Tumor Immunology Group (TIGR), Vaccine & Infectious Disease Institute (VAXINFECTIO), Faculty of Medicine and Health Sciences, University of Antwerp, 2610 Antwerp, Belgium; 2 Laboratory of Protein Science, Proteomics and Epigenetic Signaling, University of Antwerp, 2610 Antwerp, Belgium; 3 Laboratory of Membrane Trafficking, VIB-KU Leuven Center for Brain & Disease Research, 3000 Leuven, Belgium; 4 Laboratory of Experimental Hematology, Experimental Cell Transplantation Group (ECTG), Faculty of Medicine and Health Sciences, University of Antwerp, 2610 Antwerp, Belgium; 5 Immunology Laboratory, IRCCS Ospedale Policlinico San Martino, 16132 Genova, Italy; 6 Division of Hematology, Antwerp University Hospital, 2650 Edegem, Belgium; 7 Center for Oncological Research (CORE), Faculty of Medicine and Health Sciences, University of Antwerp, 2610 Antwerp, Belgium

**Keywords:** CD56 homodimers, common gamma-chain family, interleukin-15 signaling, NCAM-120, tumor cell eradication

## Abstract

A particularly interesting marker to identify anti-tumor immune cells is the neural cell adhesion molecule (NCAM), also known as cluster of differentiation (CD)56. Namely, hematopoietic expression of CD56 seems to be confined to powerful effector immune cells. Here, we sought to elucidate its role on various killer immune cells. First, we identified the high motility NCAM-120 molecule to be the main isoform expressed by immune cells. Next, through neutralization of surface CD56, we were able to (1) demonstrate the direct involvement of CD56 in tumor cell lysis exerted by CD56-expressing killer cells, such as natural killer cells, gamma delta (γδ) T cells, and interleukin (IL)-15-cultured dendritic cells (DCs), and (2) reveal a putative crosstalk mechanism between IL-15 DCs and CD8 T cells, suggesting CD56 as a co-stimulatory molecule in their cell-to-cell contact. Moreover, by means of a proximity ligation assay, we visualized the CD56 homophilic interaction among cancer cells and between immune cells and cancer cells. Finally, by blocking the mitogen-activated protein kinase (MAPK) pathway and the phosphoinositide 3-kinase (PI3K)–Akt pathway, we showed that IL-15 stimulation directly led to CD56 upregulation. In conclusion, these results underscore the previously neglected importance of CD56 expression on immune cells, benefiting current and future immune therapeutic options.

## 1. Introduction

Cluster of differentiation (CD)56 or the neural cell adhesion molecule (NCAM) is expressed in nearly all tissues. Depending on the cell type and its surroundings, three main isoforms can be generated from one single gene by alternative splicing. Based on their apparent molecular weights, they are termed NCAM-120, NCAM-140, and NCAM-180. Whereas NCAM-140 and NCAM-180 are transmembrane proteins, NCAM-120 is glycosylphosphatidylinositol (GPI)-anchored with no intracellular residues [1]. The highest CD56 levels are found in the central and peripheral nervous system, where it plays an important role mediating intracellular signal transduction and stabilizing synaptic contacts [2]. By contrast, the function of the CD56 protein in the hematopoietic system is largely undetermined, but its expression seems to be confined to activated immune cells exhibiting some level of cytotoxic properties, as recently reviewed by Van Acker et al. [3]. Although known for its role as the archetypal phenotypic marker of natural killer (NK) cells [4], CD56 can actually be expressed by many more immune cell subsets including NKT cells, gamma delta (γδ) T cells, CD8 T cells, monocytes, dendritic cells (DCs) [3], and cytokine-induced killer (CIK) cells [5]. Alluding to a central role of the CD56^+^ immune cell fraction, both functional and numerical aberrations affecting this subset are observed in a wide variety of pathologies, comprising several malignant diseases [6,7,8,9,10,11]. 

Ranked third on the cancer immunotherapy trials network (CITN) priority list of immunotherapy agents, the pleiotropic cytokine interleukin (IL)-15 has a lot of potential to boost the anti-tumor immune response and help cure cancer [12,13,14]. This is also reflected by the results of the first in human clinical trial of recombinant IL-15 [15] and of the IL-15 superagonist complex ALT-803 [16], both showing a favorable safety profile together with proliferation and activation of NK cells and T cells. Interestingly, CD56 was found to be expressed by our next-generation IL-15 DC vaccine, differentiated with IL-15 instead of IL-4, and was absent on conventional IL-4 DCs [17]. In turn, IL-15 DCs secrete IL-15 themselves [18]. Natural killer cells activated by IL-15 DCs significantly upregulate CD56, both on the CD56^bright^ and CD56^dim^ NK cell subpopulations [19]. In addition, CD56 expression is significantly higher on IL-15- and isopentenyl pyrophosphate (IPP)-stimulated γδ T cells as compared to IL-2/IPP-stimulated or unstimulated γδ T cells [20]. We, therefore, hypothesize that IL-15 signaling augments CD56 expression, which is further supported by data from other groups [21,22]. For example, IL-15 can convert central memory γδ T cells into potent CD56^+^ effector cells with the ability to rapidly produce large amounts of interferon-γ and kill tumor cells [23]. Correia et al. [24] reported that after 12 days of culture with IL-15, purified CD8^+^CD56^−^ T cells expressed de novo CD56. It is known that IL-15 is capable of enhancing the cytotoxic effector functions of lymphocytes through three pathways, i.e., (1) the Janus kinase (JAK)–signal transducer and activator of transcription (STAT) pathway; (2) the mitogen-activated protein kinase (MAPK) pathway, and (3) the phosphoinositide 3-kinase (PI3K)–Akt pathway [25]. To advance our understanding of the individual contribution of each pathway to the enhanced activation and cytotoxic state of the γδ T cells and their expression of CD56, all three pathways were blocked separately.

An initial impetus pursuing the clarification of the upregulation and functional role of CD56 on immune cells was taken in this paper. In the first part, we looked at the CD56 phenotype and isoforms of different immune cell subsets on the molecular and protein level. Next, we investigated if CD56 was actively involved in the cytotoxic capacity of CD56^+^ effector cells by means of neutralizing CD56 in a killing assay against a panel of CD56^+^ tumor cell lines. Furthermore, we looked for definitive proof of the existence of a CD56–CD56 interaction on the surface of immune cells and tumor cells by means of Duolink® Technology (Merck; Overijse, Belgium). In the second part, we examined if IL-15 and, more broadly, cytokines of the common gamma-chain cytokine family play a central role in the activation of immune cells and their upregulation of CD56.

## 2. Results

### 2.1. CD56 Isoform Expression in Immune Cells

**Figure 1:**
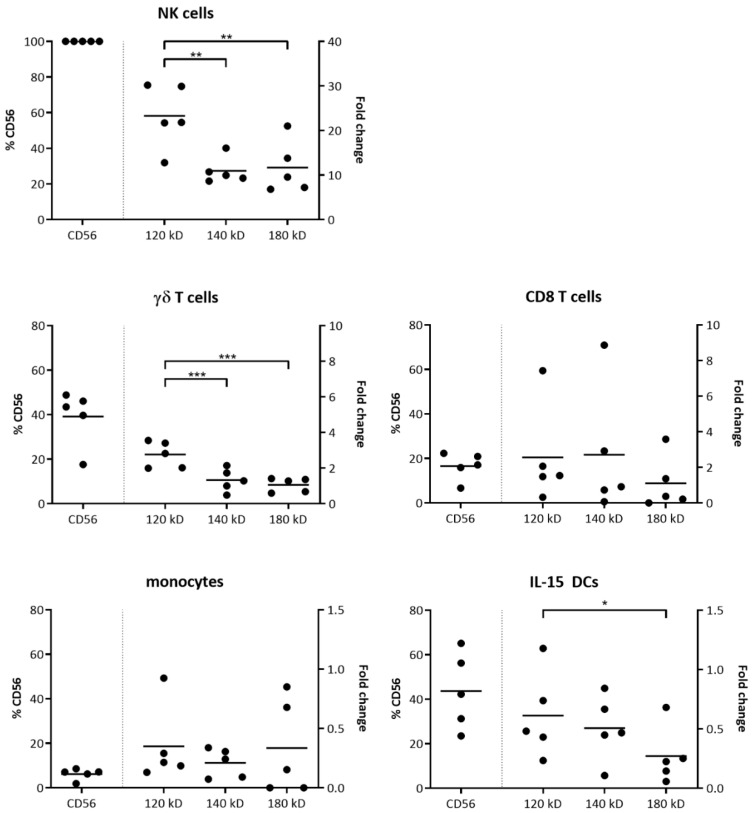
Cluster of differentiation (CD)56 (isotype) expression by different immune cell subsets. Juxtaposition of the percentage CD56 expression on different immune cell subsets as determined by flow cytometry (left *y*-axis) and CD56 isoform (120–140–180 kD) mRNA expression (scaled to average) determined by qPCR (right *y*-axis). Data from five donors are represented as scatter plots. One-way ANOVA with Bonferroni’s multiple comparison test or Friedman test with Dunn’s multiple comparison test was used. *** *p* < 0.001; ** *p* < 0.01; * *p* < 0.5.

Expression of CD56 was found on a variety of immune cells (Figure 1). Considering human NK cells are generally characterized by a CD56^+^CD3^−^ phenotype, all NK cells express some level of CD56. A significant portion of γδ T cells (39.14 ± 6.86%), CD8^+^ T cells (16.59 ± 2.73%), monocytes (6.19 ± 1.14%), and monocyte-derived IL-15 DCs (43.64 ± 7.68%) express CD56 as well. In view of the existence of several isoforms of CD56, generated by alternative splicing and posttranslational modifications, we identified the subsets expressed by the different immune cells on the molecular level by quantitative-PCR (qPCR). Protein expression validations and specific amplification of CD56 isoforms by qPCR were performed on immune cells of the same donors (*n* = 5). Interestingly, NK cells and γδ T cells favored the expression of NCAM-120 over the two transmembrane proteins NCAM-140 and NCAM-180 (Figure 1). This preference for the high motility 120 kD CD56 isoform was also seen with the IL-15 DCs, although the 140 kD isoform assumed a higher share on this immune cell subset as compared to NK cells and γδ T cells. CD8 T cells and monocytes did not prioritize the expression of one of the three isoforms.

### 2.2. Involvement of CD56 in Immune Effector Cell Activation and CD56^+^ Tumor Cell Killing

**Figure 2:**
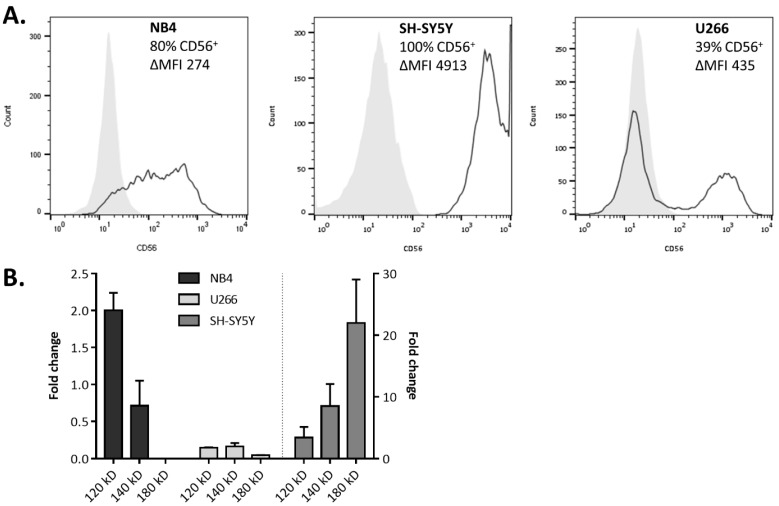
Cluster of differentiation (CD)56 expression by human tumor cell lines. (**A**) Flow cytometric analysis of tumor cells labelled with CD56-PE (black line) or corresponding isotype control (filled grey), represented as histogram overlays. (**B**) Real-time qPCR data of the expression levels of the different CD56 isoforms by NB4, U266 (left *y*-axis) and SH-SY5Y (right *y*-axis). Fold-changes are scaled to average. Each bar is the mean ± standard error of the mean (SEM) of technical replicates (*n* = 2).

**Figure 3:**
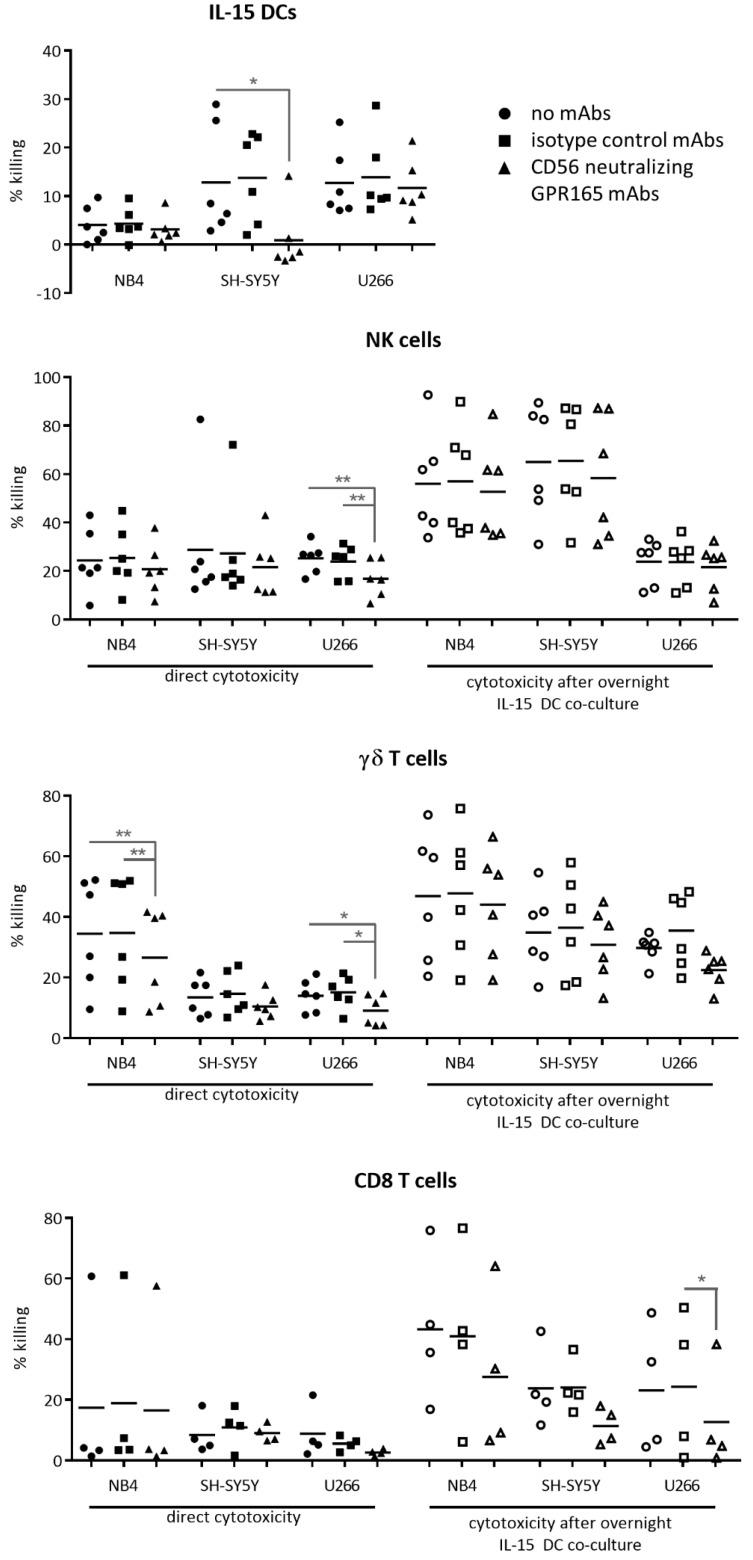
Involvement of Cluster of differentiation (CD)56 in immune effector cell activation and tumor cell killing. Immune cell cytotoxicity was defined against the cell lines NB4, SH-SY5Y, and U266, unstimulated and after overnight culture with interleukin (IL)-15 dendritic cells (DCs). Immune cells were cultured in medium without neutralizing monoclonal antibodies (mAbs) (circles) or in medium containing either CD56 neutralizing GPR165 mAbs (triangles) or its corresponding isotype control (squares) (*n* = 4–6, two independent experiments). One-way ANOVA with Bonferroni’s multiple comparison test or Friedman test with Dunn’s multiple comparison test. ** *p* < 0.01; * *p* < 0.5.

Next, we tested the cytotoxic capacity of the different CD56-expressing immune cell subsets against a panel of CD56^+^ tumor cell lines (Figure 2). As members of the innate immune system, empowered with major histocompatibility complex (MHC)-independent cytolytic capacity, unstimulated NK cells and γδ T cells were able to kill the CD56^+^ tumor cell lines NB4, SH-SY5Y, and U266 to a variable degree (Figure 3, left panels), while unstimulated CD56-enriched CD8 T cells only showed marginal killing. At an effector: target cell (E:T) ratio of 20:1, the IL-15 DC vaccine manifested its “killer-like” DC profile as well, especially against SH-SY5Y (12.81 ± 4.65%) and U266 (12.72 ± 2.95). Importantly, direct cytotoxicity of IL-15 DCs, NK cells, and γδ T cells was modulated by the addition of anti-CD56 blocking monoclonal antibodies (mAbs) to varying degrees, depending on the target cell line used (Figure 3, right panels). This suggests, at least in part, the involvement of CD56 in the lysis of malignant CD56-expressing cells. Surprisingly, we observed a strong enhancement of the killing capacity of enriched CD56^+^ CD8 T cells by IL-15 DCs. Tumor cell-killing by unprimed CD8 T cells co-cultured overnight with IL-15 DCs was 2–3 fold enhanced against NB4, SH-SY5Y, and U266 cells, i.e., 17.43 ± 14.45% →  43.32 ± 12.32%, 8.46 ± 3.27% → 23.87 ± 6.62%, and 8.82 ± 4.35 → 23.17 ± 10.61%, respectively. Upon CD56 neutralization, the lytic activity of IL-15 DC-primed CD8 T cells was reduced to levels comparable to that of unstimulated CD8 T cells. Concerning NK cells and γδ T cells alike, a clear enhancement in tumor cell killing was seen after overnight co-culture with IL-15 DCs against two out of three tumor cell lines tested. The role of CD56 in innate effector cell activation by IL-15 DCs was, however, less pronounced as for the CD8 T cells. This observed cell type specificity may be related to the effects of both CD56 and IL-15 DCs.

### 2.3. Proximity Ligation Assay Reveals Homophilic CD56 Binding between CD56-Expressing Immune and Tumor Cells

**Figure 4:**
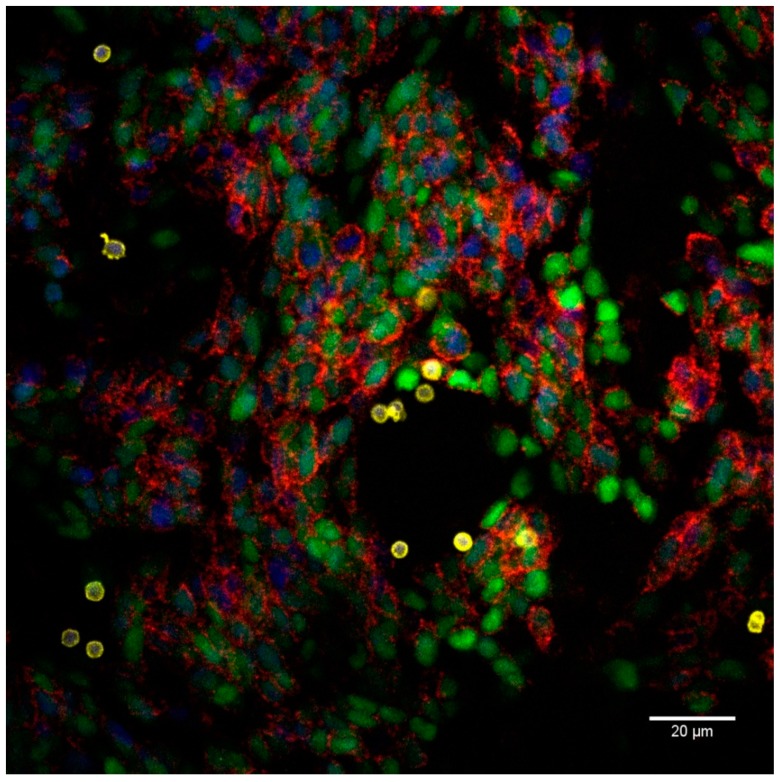
Image of a Duolink® in situ proximity ligation assay (PLA) showing Cluster of differentiation (CD)56 homodimer formation among tumor cells. Representative example of a SH-SY5Y-eGFP (green) culture (with natural killer cells (CD45; yellow)), mounted with 4′,6-diamidino-2-phenylindole (DAPI) (blue), whereby the CD56–CD56 interaction is visualized as red dots (*n* = 9). Scale bar: 20 µm.

**Figure 5:**
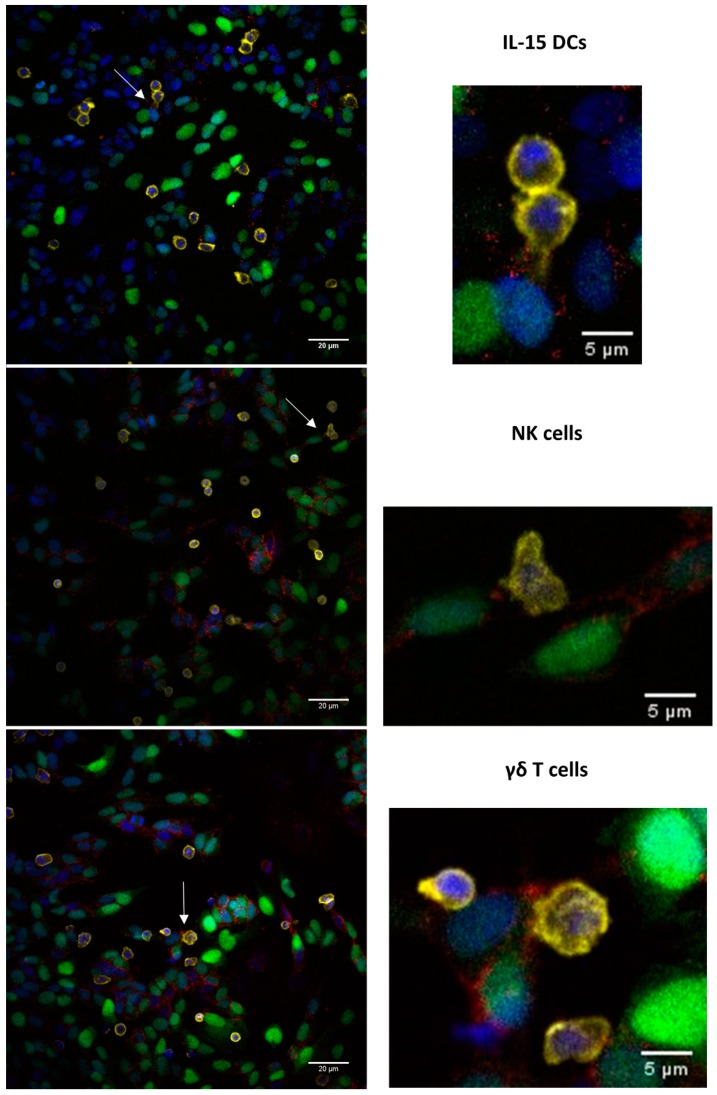
Association of Cluster of differentiation (CD)56–CD56 interactions and the “kiss of death”—cytotoxic immunological synapse between immune cells and cancer cells. SH-SY5Y-eGFP cells (green) were co-cultured with immune effector cells (CD45; yellow), namely, interleukin (IL)-15 dendritic cells (DCs) (upper row, *n* = 3), natural killer cells (middle row, *n* = 3) or γδ T cells (bottom row, *n* = 3), and mounted with 4′,6-diamidino-2-phenylindole (DAPI) (blue). White arrows on the left overview images indicate a probable interface between immune cells and cancer cells (enlarged in the right column). The connection between both cell types is associated with an accumulation of red signal, being CD56 homodimerization. Scale bar: left column = 20 µm, right column = 5 µm.

Subsequently, we sought to identify whether a homophilic CD56–CD56 binding was formed between CD56^+^ cells. To visualize this protein interaction, we performed a proximity ligation assay (PLA) analysis. From all the cultures tested, it was apparent that neuroblastoma cells, namely, SH-SY5Y-eGFP cells, strongly interacted with one another via CD56 homodimerization, not least in areas of cell confluency (Figure 4). The CD56^+^ immune cells (IL-15 DCs, NK cells, and γδ T cells) were captured too interacting with CD56^+^ tumor cells by means of a CD56 homophilic binding, whereby an accumulation of red signal (CD56–CD56 interaction) was observed (Figure 5). The interaction rate was lower as between neuroblastoma cells, likely due to the CD56^+^ immune cells’ lower mean fluorescence intensity (MFI) of CD56 expression and their non-adherent nature. 

### 2.4. IL-15 Stimulation Directly Leads to CD56 Expression 

**Figure 6:**
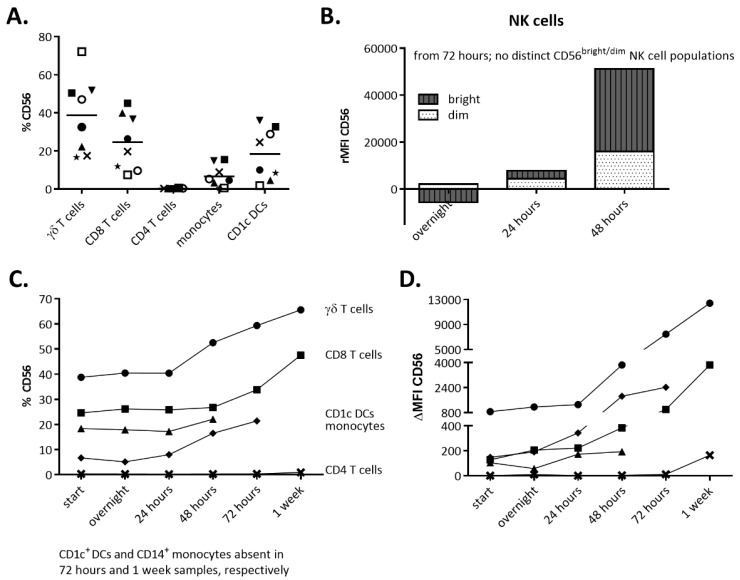
Cluster of differentiation (CD)56 expression and kinetics following interleukin (IL)-15 stimulation. (**A**) CD56 surface expression on different immune cell subsets in fresh peripheral blood mononuclear cells (PBMCs) as determined by flow cytometry. Donors are represented by unique symbols (*n* = 8). (**B**–**D**) PBMCs (1 × 10e^6^ cells/mL) were stimulated with 10 ng/mL recombinant IL-15. At different time points, the expression of CD56 was assessed flow cytometrically. (**B**) CD56 expression levels in mean fluorescence intensity (MFI), of CD56^bright^ and CD56^dim^ NK cells, were transformed to relative levels by the subtraction of the MFI of the fresh subset (*n* = 8). (**C**–**D**) Monitoring of CD56 expression on the cell membranes of γδ T cells (circle), CD8 T cells (square), CD1c dendritic cells (DCs) (triangle), monocytes (diamond), and CD4 T cells (cross), both in % (**C**) and ΔMFI (MFI condition—MFI isotype control) (**D**). The evolution in CD56 expression is shown as a mean of 5 independent donors.

From our previously published work [17,19,20], it is apparent that exposure to IL-15 might be linked to increased CD56 expression. In preliminary titration experiments, we established that the optimal concentration of IL-15 for promoting CD56 expression by immune cells is 10 ng/mL (data not shown). Subsequently, the kinetics of the CD56 expression on the immune cell surface was assessed at different time points (Figure 6). Concerning NK cells, for which CD56 is a prototypic cell surface marker, we detected a steady increase over time in the number of CD56 molecules on the CD56^dim^ NK cell surface following IL-15 stimulation (Figure 6B, Figure S1). CD56^bright^ NK cells, on the other hand, initially seemed to downregulate their CD56 expression overnight, followed by an upregulation definitely visible after 48 hours of IL-15 stimulation (Figure 6B). After this time point, a clear distinction between the CD56^dim^ and CD56^bright^ NK cell subsets was no longer discernible (data not shown). Correspondingly, we also detected a clear rise in the percentage of CD56-expressing γδ T cells after an initial lag period of 24 hours after the addition of IL-15, and a concomitant increase in its density on the cell membrane (Figure 6C,D). This expansion of CD56^+^ γδ T cells continued until at least one-week post-stimulation. Concerning CD8 T cells, a more delayed yet rapid induction and upregulation in CD56 expression was evident after 72 hours of stimulation (Figure 6C,D). CD4 T cells, however, were found to be negative for CD56 and remained CD56-negative after IL-15 stimulation. A portion of CD1c^+^ myeloid DC weakly expressed CD56 at baseline and there was only a limited, if any, effect of IL-15 after 48 hours of culture with IL-15. On the other hand, CD14^+^ monocytes rapidly induced and/or upregulated their CD56 levels. However, CD14^+^ cells were no longer detectable in peripheral blood mononuclear cells (PBMCs) exceeding 72 hours of culture with IL-15.

In addition, complementary analysis of cytotoxicity-related markers (perforin, granzyme B, TRAIL, FASL, NKG2D) as well as of some activation markers (CD69, HLA-DR) was performed (Table S1). However, no direct relationships between these markers and CD56 (following IL-15-mediated stimulation) could be drawn, except for a significant difference between granzyme B-positive cells amid the CD56^+^ CD8 T cells (75.91 ± 7.81%) as compared to CD56^−^ CD8 T cells (14.33 ± 3.51%) and CD69^+^ cells amongst CD56^+^ monocytes (10.59 ± 2.58%) and CD56^−^ monocytes (2.90 ± 0.85%).

### 2.5. Blocking of the Different IL-15 Signaling Pathways 

**Figure 7:**
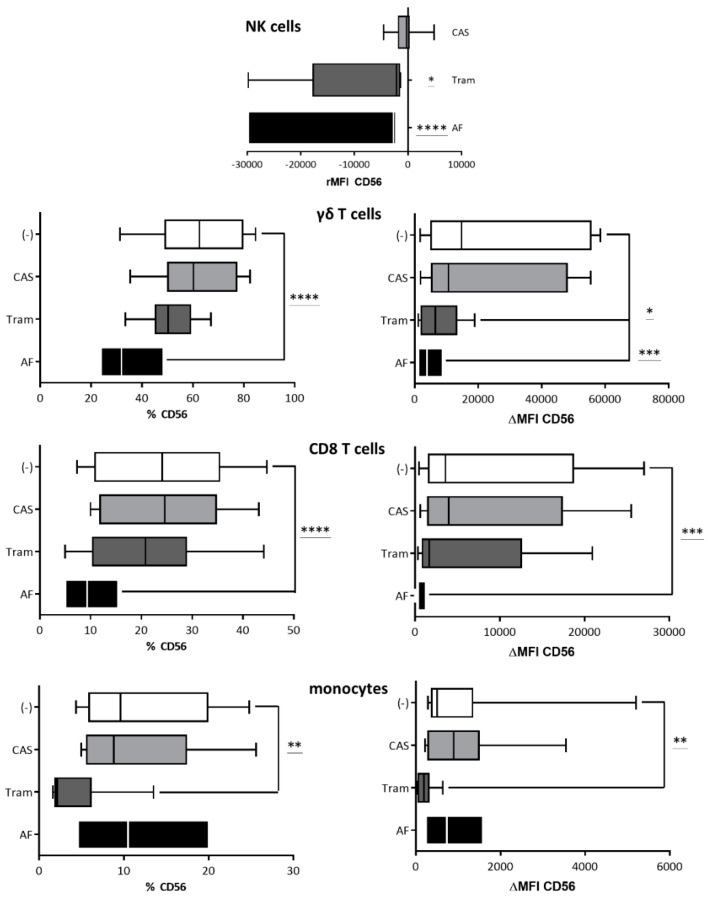
The effect of the selective inhibition of the interleukin (IL)-15 signaling pathways on Cluster of differentiation (CD)56 expression. Peripheral blood mononuclear cells (PBMCs) were cultured in IL-15 (10 ng/mL) containing medium (−) in the presence of CAS 285986-31-4 (1 µM; CAS), trametinib (1 µM; Tram) or afuresertib (2 µM; AF) for 48 hours (Natural killer (NK) cells, monocytes) or 1 week (γδ T cells, CD8 T cells). The percentage CD56-postive cells are shown, as determined by flow cytometry, as well as the ΔMFI) of CD56 calculated by subtracting the “background” MFI of the isotype control from the MFI of the sample. Concerning NK cells, the MFI of the IL-15 medium (−) control was subtracted from the MFI of the sample (rMFI). Data are represented as boxes and whiskers (10–90%) of 9 independent donors. One-way ANOVA with Bonferroni’s multiple comparison test or Friedman test with Dunn’s multiple comparison test were used. **** *p* < 0.0001; *** *p* < 0.001; ** *p* < 0.01; * *p* < 0.5.

To assess the IL-15 signaling pathway(s) involved in the induction and/or upregulation of CD56 on immune cells, the selective inhibitors CAS 285986-31-4 (JAK/STAT pathway), trametinib (MAPK pathway), and afuresertib (PI3K pathway) were used (Figure 7, Figure S2). Afuresertib drastically inhibited IL-15-mediated CD56 upregulation in NK cells, γδ T cells, and CD8 T cells. Therefore, the PI3K pathway is likely to be involved in the IL-15-mediated upregulation of CD56 expression on effector lymphocytes. Additionally, albeit to a lesser extent, trametinib significantly reduced the level of CD56 expression in NK cells and γδ T cells after IL-15 exposure (Figure 7), suggesting that the MAPK pathway of IL-15 signaling might play a role in the upregulation of CD56 expression in NK and γδ T cells. In contrast, only trametinib was able to inhibit IL-15-mediated upregulation of CD56 expression on the membrane of monocytes, suggesting distinct IL-15 signaling pathways are involved in the de novo and upregulated CD56 expression in different immune cell lineages. The effect of blocking different IL-15 signaling pathways on cytotoxicity-related molecules and activation markers can be consulted in Table S1.

### 2.6. IL-15 Shares Its CD56-Boosting Effect with IL-2 

**Figure 8:**
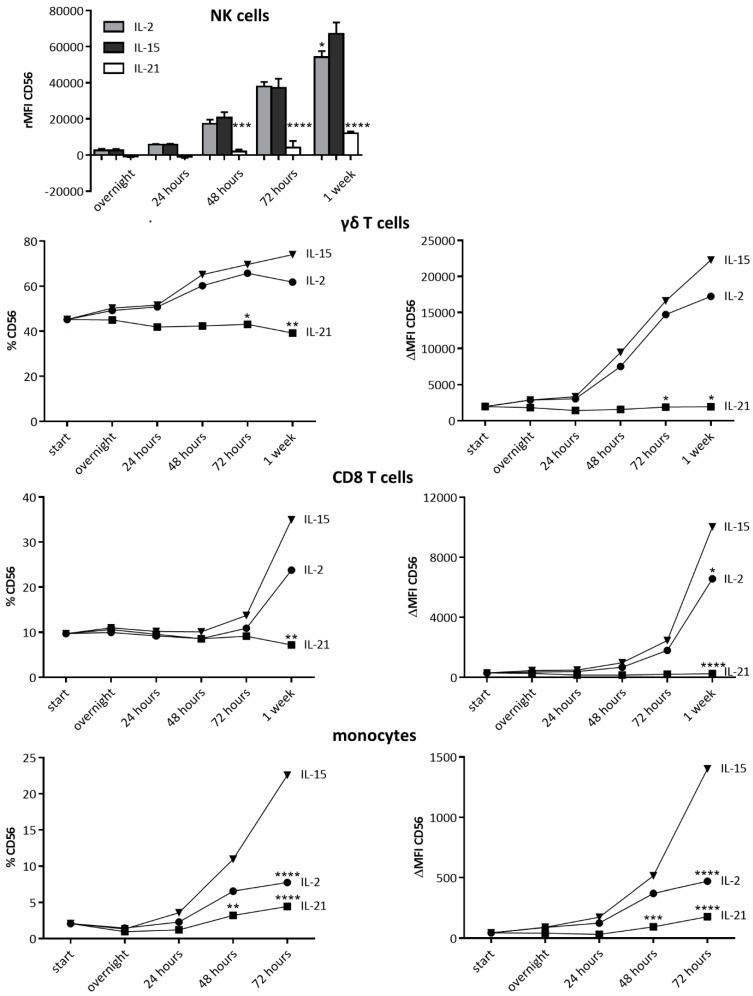
Kinetics of Cluster of differentiation (CD)56 expression following interleukin (IL)-2, IL-15, and IL-21 stimulation. Peripheral blood mononuclear cells (PBMCs) were stimulated with IL-2 (200 IU/mL), IL-15 (10 ng/mL) or IL-21 (20 ng/mL) for different periods of time, after which CD56 expression was assessed by flow cytometry (analogous to the experimental design presented in Figure 5). The evolution in CD56 expression is shown as a mean of 3 independent donors. Statistical significance is shown as compared to IL-15-stimulated immune cells. One-way ANOVA with Bonferroni’s multiple comparison test or Friedman test with Dunn’s multiple comparison test were used. **** p < 0.0001; *** *p* < 0.001; ** *p* < 0.01; * *p* < 0.5.

Given the fact that IL-15 is a cytokine of the common gamma-chain receptor family, the question arose as to whether the upregulation of CD56 by IL-15 was shared by other members of this family. Interleukin-2 (200 IU/mL) and IL-21 (20 ng/mL) were tested, as these cytokines are known to bear the highest potential for the generation of anti-cancer immune effector cells [12]. From our results, it is evident that IL-2, but not IL-21, shares the CD56-enhancing feature of IL-15, although to a lesser extent (Figure 8). Next, we assessed the contribution of the different downstream cytokine signaling pathways of IL-2 on the expression of CD56 (Figure S3). Like for IL-15-mediated CD56 induction on monocytes, the MAPK pathway is primarily involved in IL-2-induced CD56 expression. Thereto, as compared to IL-15, this pathway is also more involved in the upregulation of CD56 by IL-2 on lymphocytes. For CD8 T cells for example, the MAPK pathway and PI3K pathway are equally important, and regarding NK cells, the MAPK pathway even gets the better of the PI3K pathway. Concerning γδ T cells, no effect was actually seen with the addition of afuresertib, whereas it was manifest with the addition of trametinib. Therefore, despite the strong similarities between IL-2 and IL-15, (important) differences were seen between their signaling and effects on immune cells.

## 3. Discussion

New determinants of anti-cancer immune effector cells are warranted, enabling tumor immunologists to select and expand those cells with superior tumoricidal capacity. Here, we have demonstrated that CD56 expression on immune cells seems to be a common denominator of potent immune effector cells, endowed with cytotoxic activity against cancer cells. Moreover, CD56 itself contributes to the killing capacity of immune cells, including through the formation of homodimers. In order to induce and/or boost the expression of CD56, IL-15 stimulation can be employed, pointing to the importance of this cytokine in cellular therapy manufacturing processes. 

CD56is a known cell adhesion molecule in the neuronal system, of which the three major isoforms are called NCAM-120, NCAM-140, and NCAM-180. While the 140 and 180 kD isoforms are predominantly present in the central nervous system, extraneural human tissues mainly express NCAM-120 and NCAM-140. Accordingly, our qPCR data show very low expression levels of NCAM-120 by the SH-SY5Y cells (neuroblastoma) as compared to NCAM-180 (and NCAM-140). On the other hand, NB4/U266 (hematological malignancies) give clear preference to the NCAM-120 (and NCAM-140) isoform. Although some early reports by Lanier et al. [26,27] identified NCAM-140 as the principal isoform expressed by NK cells, it is apparent from our data that NK cells rather prefer NCAM-120. γδ T cells, innate-like killer cells, also predominantly expressed the 120 kD isoform. The difference between those findings could be attributed to the use of a human peripheral blood NK cell clone by Lanier et al. [26,27] and freshly sorted immune cells in our case. This underscores the hypothesis that the expression of the different isoforms is dependent on the cellular state and environment. In line with this, and as opposed to fresh immune cells, CIK cells were shown to express mainly the 140 kD isoform and only a small amount of the 120 kD transcript [28]. Other examples include the shift observed in neoplasia from NCAM-120 to NCAM-140 (and NCAM-180) [29,30] and the exclusive upregulation of NCAM-140 on cardiomyocytes in the context of ischemic cardiomyopathy [31]. Interestingly, epigenetic modulation has been reported to result in alternative splicing of NCAM [32].

Accordingly, functional differences can be expected from the expression of different CD56 isoforms. Whereas the transmembrane proteins NCAM-140 and NCAM-180 are known for their binding of various signaling proteins, activating a range of intracellular pathways, the GPI-anchored NCAM-120 preferentially associates with lipid rafts. As such, NCAM-120 is characterized by a high motility and a dual role as a cell-surface molecule and extracellular protein after secretion. The physiological role of shed CD56, as well as its pros and cons in the context of immunotherapy, remains to be discovered [33]. However, the higher motility of NCAM-120 could be biologically important for immune cells, facilitating cellular communication and a rapid response to external stimuli [34]. The latter being only hypothetical, since, at present, the linkage between the different isoforms expressed by the immune cells and their resulting contribution to cell functioning remains to be established. 

Looking into the overall role of CD56 in immune effector functions, we were able to establish a direct involvement of CD56 in the cytotoxic capacity of IL-15 DCs, NK cells, and γδ T cells against CD56^+^ tumor cells. It should, however, be noted that the anti-CD56 GPR165 mAb blocked the cytotoxicity of the various effector cells against the different tumor cell lines somewhat inconsistently. Jarahian et al. [35] reported something similar. Whereas some tumor cell lines transfected with NCAM-140 showed enhanced susceptibility for the cytotoxicity of IL-2-activated NK cells (i.e., PANC-1, T98G), it reduced NK cell-mediated lysis in others (i.e., LP-1, SHEP, and RPMI-8226) [35]. Thus far, no conclusive answer can be provided as to the mechanism behind these results. Furthermore, our data corroborate earlier findings of a direct role of CD56 in CIK-mediated cytotoxicity against CD56^+^ hematopoietic tumor cell targets [28]. Other indications of the functional role of CD56 in the immune system include: the CD56-mediated inhibition of tumor cell growth by IL-2-activated NK cells [36], the induction of NK cell maturation by CD56 forming a developmental synapse [37], and the role of CD56 as a pathogen recognition receptor [38]. 

Following from this, it is apparent that the number of reports investigating the functional role of CD56 on immune cells is very low. The lack of good commercially available blocking mAbs could be the cause of the scarcity of research investigating the role of CD56. Namely, the GPR165 neutralizing CD56 mAbs of Daniela Pende and Alessandro Moretta, used in this study, are the only known mAbs to effectively neutralize CD56 on human cells. Thus far, commercially available anti-CD56 mAbs, including clone B159, exhibit no CD56-neutralizing properties [28,38]. In addition, no specific neutralizing antibodies for the different NCAM isoforms have been described. Hence, tackling these limitations in future studies will provide the research community with new tools for even deeper analyses of the role of CD56’s isoforms. 

We have previously demonstrated that our IL-15 DCs are able to elicit an antigen-specific cellular immune response [39] to promote NK cell tumoricidal activity [19] and to potentiate γδ T cells to kill malignant cells [18]. The detected enhancement of NK cell- and γδ T cell-mediated killing of CD56-positive malignant cells after overnight culture with IL-15 DCs further substantiates these previous findings. However, no obvious involvement of CD56 was detected. On the other hand, as shown in Figure 3, the enhanced cytotoxic capacity of CD8 T cells (enriched for CD56) after co-culture with IL-15 DCs seems to be, at least in part, regulated by CD56 engagement. This suggests a potential crosstalk mechanism, where CD56 could function as a co-stimulatory molecule between IL-15 DCs and CD8 T cells. The specific mode of action is, however, still unknown. Indeed, besides facilitating and strengthening cell-to-cell contact, adhesion molecules like CD56 could also play a role in transmitting molecular signals across the cell membrane, regulating, in turn, a pleiotropy of cellular functions. 

Of note, although innate/unlicensed responses are generally not attributed to CD8 T cells, under certain conditions, CD8 T cells can acquire these characteristics [40,41,42]. By way of example, innate CD8 T cell activation is associated with increased immunopathology in *Leishmania* infection [43], and, more related to our current research, Sosinowski et al. [44] demonstrated the generation of functional antigen-inexperienced T cells through IL-15 trans-presentation by DCs. Interestingly, pro-inflammatory cytokine receptor signals have further been shown to use mechanisms exclusive to the T cell receptor (TCR)/CD3 signalosome to mediate bystander effector/memory CD8 T cell responses [45].

These findings raise intriguing questions regarding the nature and extent of the role of CD56 in cancer and immune cell functioning. As shown here, CD56 homodimerization is an important interaction among neuroblastoma cells. It is, therefore, likely that such connections exist among more types of CD56-expressing malignancies, including ovarian carcinomas [46], renal neoplasms [47], and small-cell lung cancer [48]. Conceivably, CD56 homodimers may be involved in the adhesion among cancer cells, and implicated in the communication and organization within the tumor micro-environment [49]. The latter could explain why CD56 expression is associated with advanced stages or dismal prognosis in different cancers [50,51,52]. On the other hand, we showed a role for CD56 in effector cell cytotoxicity and were able to provide evidence for interaction between immune cells and cancer cells via CD56–CD56 adhesion. This could account for the reports indicating a positive effect of the presence of CD56 on survival [8,53]. Altogether, CD56 expression seems to be confined to either potent immune effector cells or to malignant cancer cells, resulting in a difficult balance to manipulate in view of cancer treatment. Therapeutically eradicating CD56^+^ cancer cells could, therefore, imply incapacitating CD56^+^ cytotoxic effector cells by obstructing their connection with malignant cells. 

It is noteworthy that both cis (on the same cell surface) and trans (emanating from opposing cell surfaces) CD56 homophilic interactions can be established. While the cis homodimers do not intervene in intercellular communications themselves, they are thought to contribute to the stability of the trans interactions, mediating improved synapses with other cells [54,55]. From the PLA assay results, it is inconclusive as to whether the observed signals among the SH-SY5Y cells are all CD56 trans homodimers or whether CD56 cis homodimers are formed as well.

Finally, the question arose as to how CD56 expression is regulated and induced. There is a body of evidence showing a direct or indirect role of IL-15 signaling for upregulation of CD56: (1) culturing NK cells with IL-15 leads to an upregulation of CD56 on both CD56^bright^ and CD56^dim^ NK cells [56]; (2) IL-15 triggers the PI3K and MAPK pathways more robustly in CD56^bright^ NK cells as compared to CD56^dim^ NK cells [57]; (3) stimulation of isolated γδ T cells with IPP and IL-15 significantly upregulates CD56 levels [20]; (4) purified CD8^+^CD56^−^ T cells express de novo CD56 after 12 days of culture with IL-15 [24]; (5) umbilical cord blood T cells acquire CD56 after culture in IL-15 [58]; and (6) monocyte-derived DCs, which are differentiated with IL-15, also have a CD56-expressing subset [17,59]. In this study, we provided evidence for a direct upregulation of CD56 by IL-15, potentially through the recruitment of shc, binding to a phosphotyrosine residue on the IL-2/15Rβ chain, activating the MAPK and/or the PI3K–Akt pathway [25]. This could explain why IL-2 stimulation also leads, although at a lower level, to CD56 expression and why no such effect is observed with IL-21. Namely, the IL-2 and IL-15 receptors not only share the common gamma-chain, but also the IL-2/15Rβ chain, contrary to IL-21 [12]. From this it can be deduced that IL-15 can induce CD56 expression on immune cells bearing the IL-2/IL-15Rβ subunit. However, it is unlikely that IL-15 (and IL-2) is the sole trigger for CD56 upregulation [60]; nonetheless, it is an attractive and straightforward approach to induce immune cell activation and CD56 expression. 

In conclusion, this study identified a variable expression profile of CD56 on fresh and cultured human lymphocytes and monocytes, whereby the NCAM-120 isoform was more commonly expressed than previously presumed. Secondly, the presence of CD56 on the surface of effector immune cells was more than a mere activation marker, being directly linked to their cytotoxic capacity and immunostimulatory effect. Furthermore, we described and visualized a CD56 homophilic interaction among cancer cells themselves as well as between immune cells and malignant cells. Finally, in order to induce and upregulate CD56 expression on immune cells, we substantiated the potency of the pleiotropic cytokine IL-15, which occurred mainly through the PI3K pathway. 

## 4. Materials and Methods

### 4.1. Cell Lines and Primary Cells

This study was approved by the Ethics Committee of the Antwerp University Hospital (Edegem, Belgium) under the reference number B300201419756 on 27th January 2014. The acute myeloid leukemia cell line NB4 was purchased from the Deutsche Sammlung von Mikroorganismen und Zellkulturen (Braunschweig, Germany). The SH-SY5Y and SH-SY5Y-eGFP [61] neuroblastoma cell lines were kindly provided to us by the laboratory of Prof. Dewilde (University of Antwerp, Belgium) and the multiple myeloma cell line U266 was a gift from Prof. Germeraad (Maastricht University, The Netherlands). Peripheral blood mononuclear cells were isolated from buffy coats of healthy donors, provided by the Red Cross donor center (Red Cross-Flanders, Mechelen, Belgium). Immune cell subsets for downstream molecular biology applications were sorted on a FACSAria II flow cytometer (BD; Erembodegem, Belgium) based on the following markers within the viable cell population (Live/Dead® Fixable Aqua Stain; Thermo Fisher Scientific, Waltham, MA, USA); NK cells (CD3-PerCP-Cy5.5^−^ (BD), CD56-PE^+^ (BD)), γδ T cells (CD3-PerCP-Cy5.5^+^ (BD), γδ TCR-APC^+^ (Miltenyi, Bergisch Gladbach, Germany), CD8^+^ T cells (CD3-PerCP-Cy5.5^+^ (BD), and CD8-PB^+^ (Thermo Fisher Scientific). Flow cytometric evaluation of CD56 membrane expression of the different subsets was performed at the same time. Monocytes were isolated with anti-CD14-conjugated CliniMACS microbeads (REF 272-01; Miltenyi). For cytotoxicity experiments, NK cells were isolated with an NK cell isolation kit (REF 130-092-657; Miltenyi) and CD8 T cell purification was performed with the REAlease CD8 microbead kit (Miltenyi) followed by a CD56 enrichment step with CD56 microbeads (Miltenyi). CD8 T cells were 61% CD56-positive (range 45–89%).

### 4.2. IL-15 DC Culture

Monocyte-derived IL-15 DCs were generated according to our previously reported protocol comprising a rapid (28 hour) differentiation of monocytes with granulocyte-macrophage colony-stimulating factor and IL-15, followed by a maturation step of 20 hours by triggering of the toll-like receptor-7/8 signaling pathway [18,39,62]. 

### 4.3. γδ T Cell Expansion Protocol

γδ T cells were expanded from PBMCs as previously described [20]. Briefly, PBMCs were seeded at a final concentration of 1 × 10^6^ cells/mL in Roswell Park Memorial Institute medium supplemented with 10% plasma-derived human serum (Thermo Fisher Scientific), zoledronate (5 μM; Stemcell, Cologne, Germany), IL-2 (100 IU/mL; ImmunoTools, Friesoythe, Germany), and IL-15 (10 ng/mL, ImmunoTools). Cell cultures were maintained at a cell density of 0.5–2 × 10^6^ cells/mL and replenished every 2 to 3 days with IL-2/IL-15-supplemented medium. After 14 days, γδ T cells used in functional assays were on average 94% pure and 57% positive for CD56. The two donors used for the Duolink**®** assay had a mean culture purity and CD56 expression level of 96% and 45%, respectively. 

### 4.4. RNA Extraction and Real-Time Polymerase Chain Reaction

Total RNA was extracted from 250,000 cells using the RNeasy Micro Kit (Qiagen, Hilden, Germany), according to the manufacturer’s instruction. Subsequent quantification and purity evaluations were performed on a NanoDrop spectrophotometer (Thermo Fisher Scientific). First-strand cDNA synthesis was conducted with the iScript cDNA Synthesis Kit (Bio-Rad, Hercules, CA, USA), of which 10 ng was taken for each qPCR analysis. SYBR Green technology was used for relative mRNA quantification by qPCR on a CFX96 C1000 thermal cycler (Bio-Rad). The PrimePCR cycling protocol consisted of an initial pre-incubation of 30 s at 95 °C, followed by 40 cycles of 95 °C for 5 s and 60 °C for 30 s. The following primers were used to amplify CD56 isoform specific transcripts: *120 kD CD56* (F-CACAGCCATCCCAGCAACCTTG, R-TCCAAAGGGGGCACTGATCTT), *140 kD CD56* (F-CACTGACGGAGCCCGAGAAG, R-TCATGCTTTGCTCTCGTTCTCC), *180 kD CD56* (F-GACCCCAGATATTGACCTTGC, R-CCTTCTCGGTCTTTGCTGGC). The primers of the CD56 isoforms have been described previously [28]. Three housekeeping genes (*B2M*, *SDHA,* and *TBP*) were carefully chosen from a list of 8 reference targets, also including *ACTB*, *IPO8*, *PGK1*, *RPL13,* and *RPS14*, using geNorm in qBase+ (geNorm V < 0.15; Version 2.6.1, Biogazelle, Gent, Belgium). Inter-run calibrators were included. All primers were purchased from Bio-Rad. Fold-changes were calculated using the comparative Cq method, scaled to average.

### 4.5. Cytotoxicity Assay

Determination of the (innate-like) cytotoxic capacity of different immune cells was performed by means of a 4 hour flow cytometry-based lysis assay against PKH67-labeled (Thermo Fisher Scientific) CD56^+^ cell lines; NB4, SH-SY5Y, and U266 (Figure 2). To specify the direct killing capacity of isolated NK cells, CD8 T cells, and expanded γδ T cells, they were co-cultured at an E:T ratio of 5:1, whereas IL-15 DCs were cultured at an E:T ratio of 20:1. To investigate the involvement of CD56, 100 µL of the neutralizing GPR165 mAbs-containing supernatant (IgG2a) [28,38] was added to specific conditions. To assess the involvement of CD56 in immune effector cell activation by (CD56^+^) IL-15 DCs, NK cells/γδ T cells/CD8 T cells were cultured overnight at a 1:1 E:T ratio with IL-15 DCs, whether or not in the presence of anti-CD56 neutralizing antibodies or isotype controls. Subsequently, cultures were washed and PKH67-positive target cells were added at a 5:5:1 ratio for four hours. Target cell death was determined by combined annexin-V-APC (BD)/propidium iodide (PI; Merck) staining. Specific target cell killing was calculated using the following formula: % killing = 100−((% annexin-V^–^PI^–^ target cells with effector cells/% annexin-V^–^PI^–^ target cells without immune cells) × 100).

### 4.6. Blocking of the Distinct IL-15 Signaling Pathways 

Iinterleukin-15 signaling pathways were blocked using either (1) CAS 285986-31-4 (1 µM; Merck) as a STAT5 Inhibitor, (2) trametinib (1 µM; GSK1120212; Selleckchem, Munich, Geramany) as a highly selective inhibitor of both MEK1 and MEK2, and (3) afuresertib (2 µM; GSK2110183, Selleckchem) as a highly selective inhibitor of Akt. Concentrations of the different inhibitors were first titrated to ascertain preservation of cell viability. One hour prior to the addition of IL-15 (10 ng/mL), PBMCs (1 × 10^6^ cells/mL) were exposed to the abovementioned inhibitors to ensure adequate blocking. Natural killercells and monocytes were analyzed after 48 h, whereas γδ T cells and CD8 T cells were assessed after 1 week. For immunophenotypic analysis, cells were stained with the following fluorochrome-labeled mAbs: CD3-FITC (Immunotools), CD8-FITC, CD14-FITC, γδ TCR-FITC (Miltenyi), CD69-PE, CD314-PE, CD3-PerCP-Cy5.5, CD56-BV421, CD3-APC, CD253-APC, γδ TCR-APC (Miltenyi), CD178-BV786, and HLA-DR-APC-H7 (or isotype controls). Fc receptor blocking was done with mouse gamma globulins (Jackson ImmunoResearch, West Grove, PA, USA) for 10 min prior to staining with mAbs. For the combined cell surface and intracellular perforin and granzyme B staining, Brefeldin A (1 µL/mL; Golgi-Plug, BD) was added to the cultures for four h prior to membrane staining. Cultures were fixed and permeabilized with the Foxp3 Transcription Factor Staining Buffer Set (Invitrogen, Ottawa, ON, Canada) and stained with granzyme B-BV421 and perforin-AF647. All mAbs were purchased from BD, unless stated otherwise. Additionally, dead cells were excluded using Live/Dead® Fixable Aqua Stain (Invitrogen).

### 4.7. Proximity Ligation Assay

To study CD56 homodimerization, Duolink^©^—proximity ligation assay (PLA) analysis was performed. Per 24 well, 150,000 SH-SY5Y-eGFP cells were plated for three days on round 12 mm cover glasses (VRW, Radnor, PA, USA). Subsequently, 750,000 immune cells, i.e., IL-15 DCs, NK cells or γδ T cells, were added to the wells and co-incubated for 20 minutes. Fixation of the co-cultures was performed with 4% formaldehyde. Goat anti-human CD56 mAbs (AF2408, 1:200; R&D, Minneapolis, MN, USA) were labeled with the Duolink® in situ probemaker PLUS or MINUS. Identification of immune cells relied on staining with CD45 mAbs (YAML501.4, 1:500; Thermo Fisher Scientific) in combination with Cy™5 AffiniPure donkey anti-rat IgG mAbs (1:100; Jackson ImmunoResearch). Samples were amplified with the Duolink® In Situ Detection Reagents Red (#DUO92008), generating red fluorescent spots if two CD56 proteins are located in a radius of 40 nm (dimerization). The cover slips were mounted with Duolink® in situ mounting media with DAPI. Images were captured on a Nikon TI-E inverted confocal microscope (Nikon, Tokyo, Japan) and analyzed with ImageJ. 

### 4.8. Statistical Analysis

All flow cytometry data were acquired on a FACSAria II flow cytometer (BD) and analyzed using FlowJo (v10; Treestar, Ashland, OR, USA). GraphPad Prism software (v7.00; Graphpad, San Diego, CA, USA) was used for statistical analysis and artwork. The Shapiro–Wilk normality test was performed to test if the datasets came from a Gaussian distribution.

## Supplementary Materials

The following are available online at https://www.mdpi.com/2072-6694/11/7/1029/s1, Table S1: Complementary analysis of activation and cytotoxicity related markers on CD56-expressing immune cells, Figure S1: CD56 expression levels on NK cells following stimulation with IL-15, Figure S2: The contribution of the different IL-15 signaling pathways on CD56 expression, Figure S3: Comparison between blocking the IL-2 and IL-15 signaling pathways.
