# Supplementary material for: CD56 Homodimerization and Participation in Anti-Tumor Immune Effector Cell Functioning: A Role for Interleukin-15
Source: Cancers (Basel). 2019 Jul 22;11(7):1029. doi: 10.3390/cancers11071029 (PMC6678613; doi:10.3390/cancers11071029)
Supplement: Supplementary file 1 [file cancers-11-01029-s001.pdf]

Supplemetary Materials

Table S1. Complementary analysis of activation and cytotoxicity related markers on CD56-expressing immune cells.

**NK cells**	CD69 ± SEM	HLA-DR ± SEM	NKG2D ± SEM	FASL ± SEM	TRAIL ± SEM
(-)	36.37 ± 9.73	13.73 ± 0.67	96.47 ± 0.61	0.17 ± 0.07	10.73 ± 5.17
CAS	21.97 ± 4.40	13.27 ± 0.78	96.57 ± 0.72	0.30 ± 0.16	11.33 ± 4.88
Tram	0.93 ± 0.39 *	15.27 ± 3.11	80.20 ± 4.45 **	0.23 ± 0.07	3.10 ± 2.00 *
AF	18.57 ± 11.56	7.50 ± 2.11	86.53 ± 4.14 *	0.20 ± 0.08	4.40 ± 2.71

**NK cells**	granzyme B ± SEM	perforin ± SEM
(-)	8.22 ± 1.75	2.75 ± 1.01
CAS	8.14 ± 1.74	5.20 ± 1.64
TRAM	7.25 ± 1.35	12.17 ± 3.32
AF	3.85 ± 1.10	9.67 ± 5.02

**γδ T cells**	CD69 ± SEM	HLA-DR ± SEM	NKG2D ± SEM	FASL ± SEM	TRAIL ± SEM
(-)	68.97 ± 8.90	56.67 ± 3.98	98.73 ± 0.29	0.02 ± 0.02	1.41 ± 0.87
CAS	65.40 ± 9.62	55.27 ± 7.70	99.30 ± 0.06	0.03 ± 0.03	3.31 ± 1.85
TRAM	4.77 ± 2.55 ***	42.22 ± 14.48	97.17 ± 0.75	0.13 ± 0.04	0.09 ± 0.08
AF	41.50 ± 1.30	38.54 ± 7.81	97.20 ± 0.96	0.03 ± 0.03	0.27 ± 0.14
**Within CD56$^+$ population**	CD69 ± SEM	HLA-DR ± SEM	NKG2D ± SEM		
	82.01 ± 4.25	55.80 ± 5.01	99.59 ± 0.20		
	77.66 ± 6.08	54.36 ± 9.58	99.75 ± 0.09		
	8.04 ± 3.50 ***	38.93 ± 14.91	97.94 ± 0.81 *		
	55.49 ± 6.47 *	34.15 ± 6.031	98.68 ± 0.31		

**γδ T cells**	granzyme B ± SEM	perforin ± SEM
(-)	95.10 ± 2.88	6.42 ± 2.65
CAS	98.30 ± 1.12	2.81 ± 1.18
TRAM	98.07 ± 1.07	1.91 ± 0.53
AF	84.37 ± 4.16 *	24.06 ± 10.13
**Within CD56$^+$ population**	granzyme B ± SEM	perforin ± SEM
	99.71 ± 0.22	9.93 ± 4.05
	99.87 ± 0.13	5.14 ± 2.72
	96.42 ± 3.58	7.43 ± 3.98
	94.52 ± 0.85	33.70 ± 14.69

**CD8⁺ T cells**	CD69 ± SEM	HLA-DR ± SEM	NKG2D ± SEM	FASL ± SEM	TRAIL ± SEM
(-)	12.86 ± 6.26	26.36 ± 2.99	95.26 ± 1.43	1.20 ± 0.39	0.48 ± 0.24
CAS	16.08 ± 7.04	30.50 ± 5.11	96.93 ± 0.49	2.06 ± 0.81	0.73 ± 0.34
TRAM	4.60 ± 4.42	19.14 ± 3.61	89.42 ± 1.89	1.04 ± 0.23	0.10 ± 0.06
AF	5.53 ± 2.25	12.77 ± 2.58	84.44 ± 4.90	0.27 ± 0.06	0.08 ± 0.06
**Within CD56⁺ population**	CD69 ± SEM	HLA-DR ± SEM	NKG2D ± SEM		
	31.70 ± 15.55	49.23 ± 11.90	96.73 ± 1.80		
	39.62 ± 19.07	49.88 ± 11.47	98.81 ± 0.61		
	21.22 ± 20.71	30.03 ± 6.35	91.15 ± 1.40		
	31.62 ± 20.06	23.95 ± 9.69	95.39 ± 3.12		

**CD8 T cells**	**granzyme B ± SEM**	**perforin ± SEM**
(-)	20.93 ± 6.90	0.54 ± 0.19
CAS	27.10 ± 2.24	0.27 ± 0.11
TRAM	43.51 ± 2.97 **	0.08 ± 0.03
AF	6.32 ± 1.63 *	1.24 ±0.25
**Within CD56⁺ population**	granzyme B ± SEM	perforin ± SEM
	75.91 ± 7.81	6.38 ± 2.66
	79.19 ± 4.67	3.25 ± 1.63
	77.67 ± 3.86	3.70 ± 1.57
	41.56 ± 5.97	15.04 ± 3.40 **
	75.91 ± 7.81	6.38 ± 2.66

**CD14⁺ cells**	CD69 ± SEM	HLA-DR ± SEM	NKG2D ± SEM	FASL ± SEM	TRAIL ± SEM
(-)	3.60 ± 1.08	99.03 ± 0.52	3.97 ± 0.56	0.70 ± 0.30	0.27 ± 0.09
CAS	3.83 ± 1.25	99.40 ± 0.32	2.97 ± 0.38	0.90 ± 0.38	1.50 ± 2.34
TRAM	0.50 ± 0.31 **	99.00 ± 0.65	3.97 ± 1.94	1.30 ± 0.10	3.37 ± 3.22
AF	1.30 ± 0.85 *	99.53 ± 0.15	1.03 ± 0.23	0.93 ± 0.53	0.47 ± 0.42
**Within CD56⁺ population**	CD69 ± SEM	HLA-DR ± SEM	NKG2D ± SEM		
	10.59 ± 2.58	96.48 ± 2.16	11.71 ± 2.611		
	10.40 ± 3.78	97.97 ± 1.13	8.393 ± 0.88		
	10.48 ± 8.21	95.16 ± 4.12	16.61 ± 3.53		
	5.39 ± 2.78	95.41 ± 3.97	9.80 ± 5.69		

**CD14⁺ cells**	granzyme B ± SEM	perforin ± SEM
(-)	3.87 ± 0.89	0.50 ± 0.14
CAS	4.37 ± 1.26	0.56 ± 0.16
TRAM	6.10 ± 1.14	0.64 ± 0.16 *
AF	1.40 ± 0.79	0.64 ± 0.22
**Within CD56⁺ population**	granzyme B ± SEM	perforin ± SEM
	5.18 ± 1.59	0.81 ± 0.13
	5.56 ± 1.56	1.46 ± 0.26
	5.24 ± 0.84	4.61 ± 3.26
	3.14 ± 1.26	2.36 ± 0.76

Abbreviations: PBMCs cultured in IL-15 (10 ng/mL) containing medium; CAS, CAS 285986-31-4; TAM, trametinib; AF, afuresertib. ***, $p < 0.001$; **, $p < 0.01$; *, $p < 0.5$, n = 3.

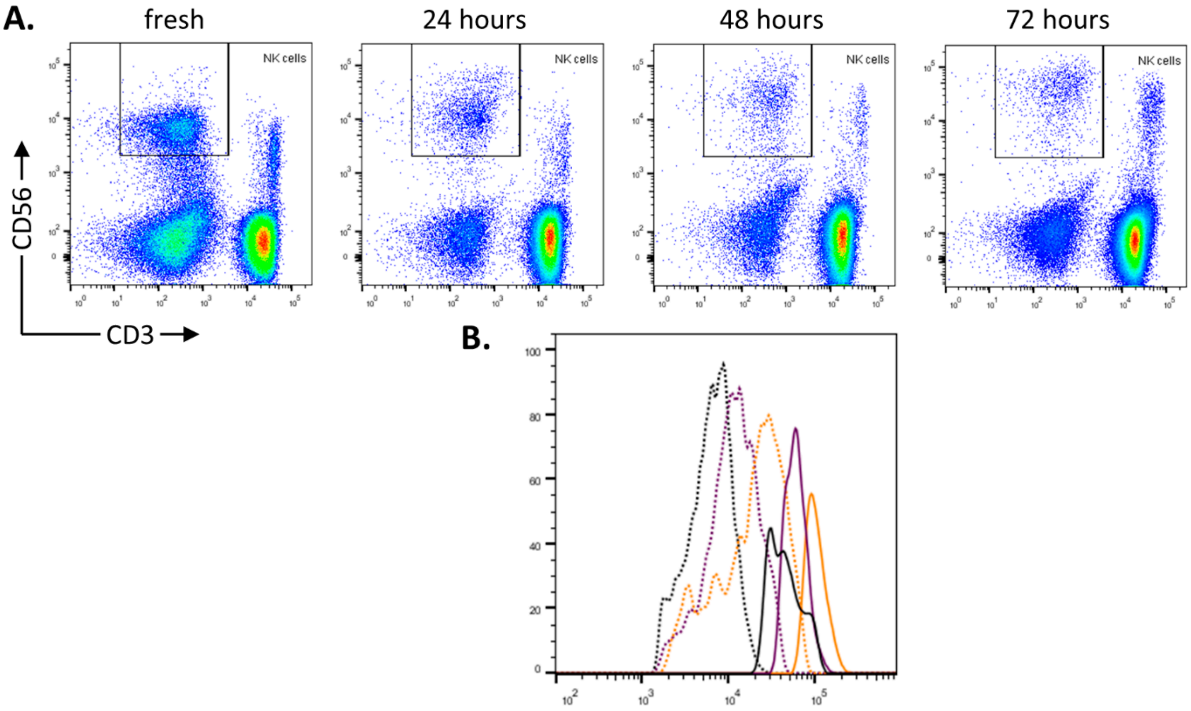

Figure S1. CD56 expression levels on NK cells following stimulation with IL-15. PBMCs (1x10e6 cells/mL) were stimulated with 10 ng/mL recombinant IL-15. **A.** Within the viable cell population, NK cells were assessed flow cytometrically at different time points. NK cell gating is shown for one representative donor. **B.** Histogram overlay of the same donor illustrating the CD56 expression on CD56^{dim} (dotted line) and CD56^{bright} (full line) NK cells; fresh (black), after 24-hour (purple) and 48-hour (orange) stimulation with IL-15.

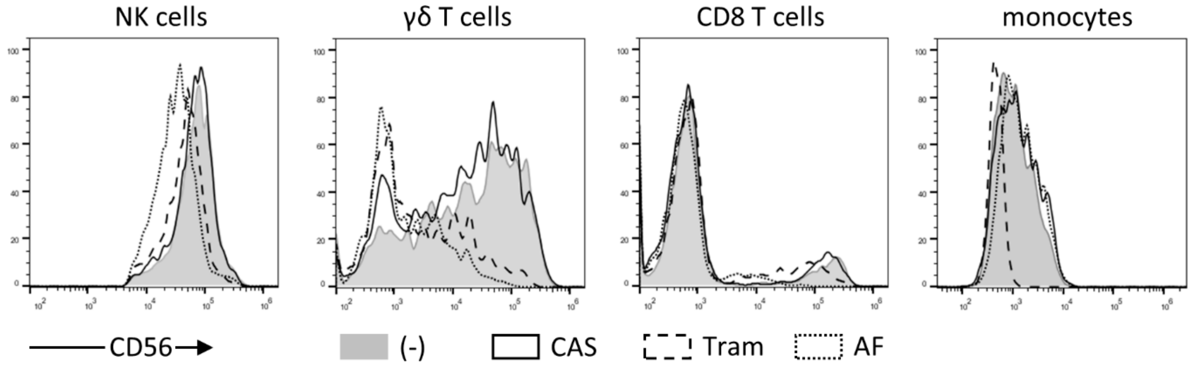

Figure S2. The contribution of the different IL-15 signaling pathways on CD56 expression. PBMCs were cultured in IL-15 (10 ng/mL) containing medium (-) in the presence of the selective inhibitors CAS 285986-31-4 (1 μM; CAS), trametinib (1μM; Tram) or afuresertib (2 μM; AF) for 48 hours (NK cells, monocytes) or 1 week (γδ T cells, CD8 T cells). The histogram overlays show PE staining levels of CD56 on different immune cell subsets.

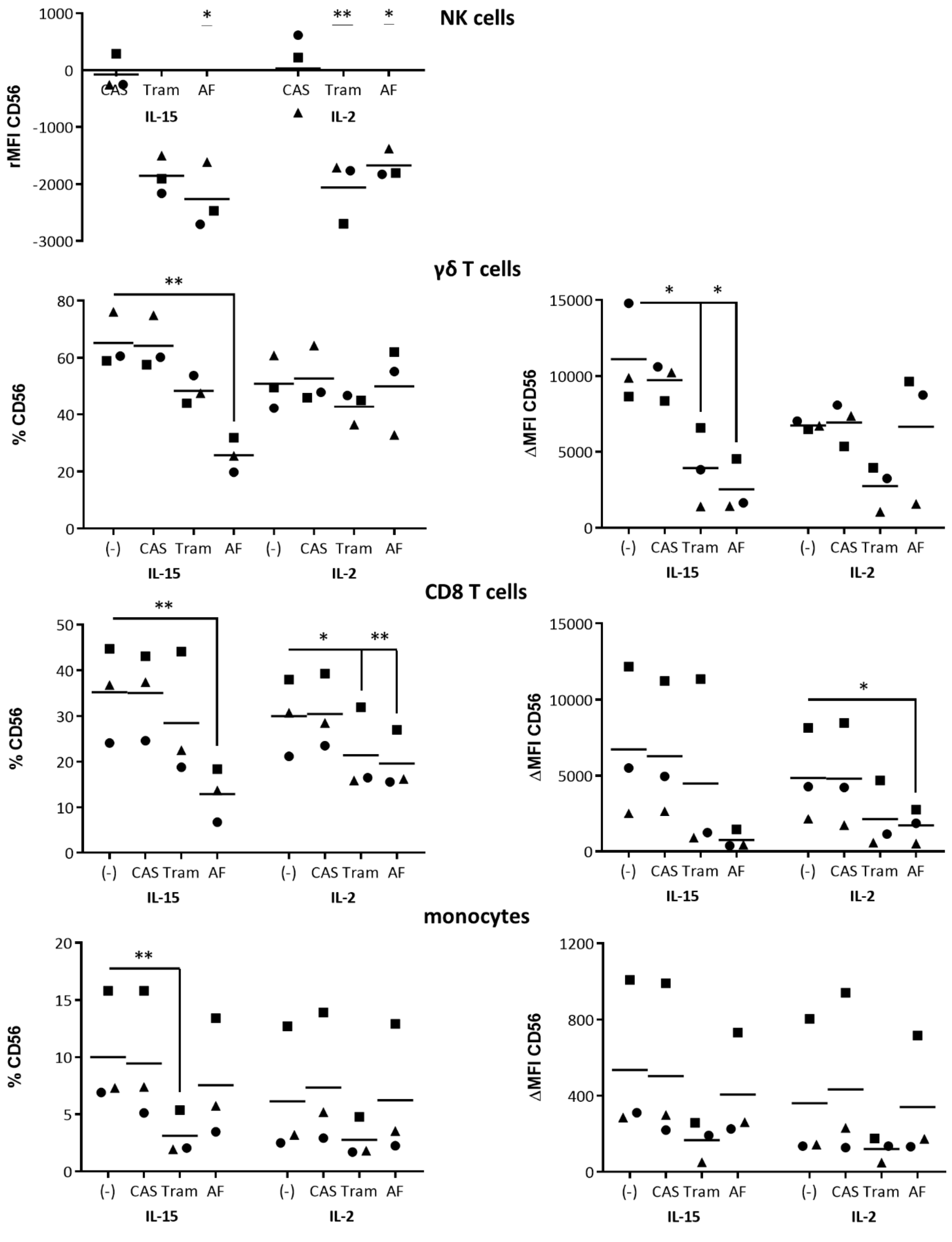

Figure S3. Comparison between blocking the IL-2 and IL-15 signaling pathways. By analogy with Figure 7, the different signaling pathways of IL-2 and IL-15 were blocked separately and assessed for their involvement in the CD56 upregulation on immune cells by their respective cytokine. One-Way ANOVA with Bonferroni's Multiple Comparison test or Friedman test with Dunn's Multiple Comparison Test. **, $p < 0.01$; *, $p < 0.05$.
